# Pharmacotherapy for Insomnia Disorder in Older Adults

**DOI:** 10.1001/jamanetworkopen.2019.18214

**Published:** 2019-12-02

**Authors:** Michael A. Grandner, Michael L. Perlis

**Affiliations:** Sleep and Health Research Program, Department of Psychiatry, University of Arizona College of Medicine, Tucson; Behavioral Sleep Medicine Program, Department of Psychiatry, Perelman School of Medicine, University of Pennsylvania, Philadelphia

Insomnia, even when variably defined, is a well-established risk factor for impaired daytime functioning, unhealthy substance use, depression, other psychiatric disorders, chronic pain, and a variety of other medical conditions, including obesity, high blood pressure, cardiovascular disease, and dementia. Accordingly, treating insomnia should be a priority, especially among older adults, as this group is at increased risk for sleep continuity disturbance (ie, problems with sleep initiation, maintenance, and duration). While the recommended first-line treatment for insomnia is cognitive behavioral therapy for insomnia,^1^ most patients will be offered or opt for medical management of their insomnia. This is true for a variety of reasons, including the unavailability of clinicians who can provide cognitive behavioral therapy for insomnia, the universal availability of pharmacotherapy, and the reduced time commitment and behavioral work required by pharmacotherapy.

Of the pharmacologic agents indicated for the treatment of insomnia disorder, nearly all of which are γ-aminobutyric acid agonists, most are associated with significant risks for use in older adults.^2^ This being the case, other medications tend to be prescribed for older adults. These include on-label approaches (eg, melatonin agonists, such as ramelteon) and off-label approaches (eg, sedating antidepressants, such as trazodone). More recently, a new class of pharmacologic agents, dual orexin receptor antagonists, has been developed, evaluated, approved, and introduced into prescriptive formularies. This new class of medications holds the promise of greater efficacy, as the target mechanism may more directly address the issue of the failure to inhibit wakefulness, and the potential for reduced adverse effects, especially in the domain of cognition, memory, and psychomotor behavior.^3^ Given the promise and potential of dual orexin receptor antagonists, it is especially important that these compounds be specifically evaluated in older adults in a manner that allows for the assessment of the efficacy and safety of this new therapeutic modality compared with standard treatments, especially with benzodiazepine receptor agonists. The study by Rosenberg and colleagues^4^ does precisely this.

Rosenberg et al^4^ present data from a placebo-controlled randomized clinical trial that evaluated 2 different doses of lemborexant compared with extended-release zolpidem in older adults with severe sleep maintenance difficulties (ie, wake-after-sleep-onset [WASO] mean of ≥60 minutes on ≥3 nights per week for the past month and a polysomnographic mean WASO of ≥60 minutes across 2 laboratory studies). It is important to note that the study also ruled out placebo responders during baseline.

The primary outcomes of the study were polysomnographic sleep latency (SL) and WASO. Compared with a 6.5-minute decrease in polysomnographic SL in the placebo group, patients treated with zolpidem exhibited a reduction in polysomnographic SL of 12.6 minutes, and patients treated with lemborexant demonstrated reductions of 16.6 minutes for the 5-mg dose and 19.5 minutes for the 10-mg dose. Compared with a 15.1-minute reduction in WASO in the placebo group, the zolpidem group had a 44.4-minute reduction, the lemborexant 5 mg group had a 50.0-minute reduction, and the lemborexant 10 mg group had a 59.6-minute reduction. When evaluated by time of night (ie, change to WASO in the second half of the night) the reductions were 7.1 minutes in the placebo group, 24.6 minutes in the zolpidem group, 30.3 minutes in the lemborexant 5 mg group, and 37.1 minutes in the lemborexant 10 mg group. Both doses of lemborexant produced significantly larger effects for polysomnographically measured SL and WASO than zolpidem. As for treatment-emergent adverse events, the authors reported that the “overall incidence of treatment-emergent [adverse events] was similar among treatment groups.”^4^

Of note, if not concern, is that sleep diary values for SL and WASO were assessed and reported to be statistically improved, but the specific values were not reported. Absent these data, one cannot know how the variables that represent the patients’ presenting concerns were affected. Specific subjective data were presented using the Insomnia Severity Index. The mean changes in scores were −6.1 points in the placebo group, −8.3 points in the zolpidem group, −7.8 points in the lemborexant 5 mg group, and −7.9 points in the lemborexant 10 mg group. The treatment groups significantly differed from the placebo group but not from each other. Interestingly, Rosenberg et al^4^ used an Insomnia Severity Index factor score for the evaluation of daytime functioning (ie, sum of items 4–7). Here again, they found that treatment groups significantly differed from the placebo group but not from each other.

The study by Rosenberg et al^4^ demonstrated that objective sleep continuity improvements, specifically polysomnographically measured SL and WASO, were larger with lemborexant, particularly for the second half of the night, and both medications positively affected daytime function. Neither medication was superior with respect to safety or subjective outcomes as measured with the Insomnia Severity Index.

There is an increasing preference toward objective outcomes in trials. However, it is problematic to implement this approach in the case of insomnia. The desire to depend on unbiased, verifiable, precise end points is laudable, but it is also potentially misguided. This is especially true when the outcome of interest is something for which there is no truly valid (vs reliable) measure. While polysomnography allows for precision in sleep-stage determination, it is not a direct measure of sleep, which is regulated subcortically.^5^ Electroencephalographic staging of wake and sleep states are often discordant with momentary or morning self-reported sleep in healthy sleepers as well as people with insomnia. This discordance may be based on polysomnography’s relative insensitivity to perceptual engagement, owing to: (1) failure to take into account β-γ frequencies,^6^ (2) undue reliance on scoring based on central and occipital derivations,^7^ or (3) temporal and spatial summation of activity from the cortical mantle, so that it cannot resolve local wakefulness in small cortical or subcortical areas.^5^ These limitations notwithstanding, a case can be made that self-report assessments, particularly sleep diaries, are simply more relevant because they allow for precise, prospective diagnosis, are commonly used to guide treatment, and are essential for the assessment of treatment response. For these reasons, self-report, not objective measures, is generally recommended for the clinical evaluation and treatment of insomnia.^8^

Objectively measured improvement, in the absence of perceived improvement, is tantamount to no improvement. For example, consider chronic pain. Even if an objective measure of nociception existed, a treatment that changed this outcome without producing a diminution in the experience of self-reported pain intensity would likely be considered ineffective by the patient and the clinician. Ultimately, it is the patient’s experience of pain and treatment-related diminution of pain that is paramount. The same is true for insomnia.

A possible ideal strategy for assessing insomnia outcomes would be to deploy both objective and subjective methods and to present such data side-by-side. This is especially important given that self-report is the basis of insomnia diagnosis, assessment of treatment progression, and determination of treatment outcome.^5,8^ Furthermore, patient preferences for perceivable treatment effects and reduced adverse effect profiles might ultimately determine which medications are most successful in real-world clinical applications.
